# Latarjet procedure enables 73% to return to play within 8 months depending on preoperative SIRSI and Rowe scores

**DOI:** 10.1007/s00167-021-06475-1

**Published:** 2021-03-20

**Authors:** Yoann Bohu, Pierre Abadie, Floris van Rooij, Luca Nover, Société Française de Traumatologie du Sport, Jean Kany, Philippe Colotte, François Kelberine, Didier Fontes, Charles Edouard Thelu, Matthieu Sanchez, Julien Berhouet, Alexandre Hardy

**Affiliations:** 1 Clinique du Sport Paris V, Ramsay Santé, Paris, France; 2 Clinique du Sport de Bordeaux-Merignac, Mérignac, France; 3 ReSurg SA, 22 Rue Saint Jean, 1260 Nyon, Switzerland; 4 Service d’Orthopédie Hôpital Trousseau CHRU, Tours, France; 5 Ambroise Paré Hospital, Boulogne-Billancourt, France

**Keywords:** Latarjet, Anterior shoulder instability, Return to play

## Abstract

**Purpose:**

Systematic reviews report return to play (RTP) within 5.8 months (range, 3–8) following the Latarjet procedure, but the factors that influence RTP remain unknown. The present study aimed to report the rate and time of return to play (RTP) during the first 8 months following the Latarjet procedure, and to determine the influence of sport type or patient characteristics.

**Methods:**

The authors retrospectively collected the records of patients that underwent Latarjet procedures for anterior shoulder instability between 2015 and 2017. Patients were excluded if they had any concomitant rotator cuff tendon lesions, or previous ipsilateral shoulder surgery. The authors retrieved patient demographics, time from injury to surgery, type of sport practiced (overhead/non-overhead, contact/non-contact), as well as pre- and postoperative Western Ontario Shoulder Instability index (WOSI), Shoulder Instability-Return to Sport After Injury index (SIRSI), and Rowe score.

**Results:**

A total of 217 patients (217 shoulders) were eligible for inclusion, comprising 184 males and 33 females, aged 26.8 ± 7.3 years at index surgery. The main sport practiced prior to surgery involved overhead (*n* = 173, 80%) and/or contact (*n* = 152, 70%) activities. By 8 month follow-up, 158 patients (73%) resumed their main sport, at a mean of 5.1 ± 1.5 months. Multivariable analysis revealed that RTP was more likely in patients with higher preoperative Rowe score (OR, 1.02; *p* = 0.024) and SIRSI score (OR, 1.02; *p* = 0.008).

**Conclusions:**

By 8 months following the Latarjet procedure, 73% of patients had resumed their main sport. The likelihood of RTP was significantly associated with preoperative Rowe and SIRSI scores, but not with sport type.

**Level of evidence:**

IV.

## Introduction

Anterior shoulder instability often requires surgical treatment, particularly in young athletes that wish to resume sports [5, 29, 31]. The most common surgical procedures involve arthroscopic labral or capsular repair [27, 32], and open or arthroscopic bone-block procedures [9, 21], which transfer the coracoid process through a subscapularis split to the anteroinferior margin of the glenoid [12, 40].

The Latarjet technique is a bone-block procedure that gained considerable popularity in recent years, as it proved effective at preventing recurrent dislocations [2, 8, 19], while allowing rapid recovery and return to play (RTP) [3, 8, 19, 23]. The Latarjet procedure has also demonstrated satisfactory long-term outcomes in young recreational and competitive athletes [5, 13, 20, 41], despite comparatively high complication rates, including pain due to soft-tissue impingements [17], neurologic impairments, and bone-block nonunion or malposition [11, 13, 15, 18, 25, 39].

One of the main concerns for a young athlete undergoing surgery is if and when they will be able to RTP, notably resuming their main sport at preinjury or preoperative level. Recent studies revealed that up to 50% of athletes are able to RTP within 6 months following the Latarjet procedure [10, 14, 30], though it remains unclear whether RTP depends on the type of sport or on other demographic and surgical factors. The purpose of this study was to: (i) report the rate and time of RTP during the first 8 months following primary Latarjet procedures performed to treat anterior shoulder instability; and (ii) determine whether the rate and time of RTP are associated with type of sport or patient characteristics. The hypothesis was that the rate and time of RTP are associated with neither type of sport nor patient characteristics.

## Materials and methods

The authors retrospectively collected the records of patients that underwent Latarjet procedures over a 2-year period (July 2015–June 2017) at 9 centers. All patients provided informed consent for their participation in this study, which had been approved by the institutional review board in advance (IRB# COS-RGDS-2020–05-022-BOHU-Y, Ramsay Santé Comité d’Ethique; + 33 (0)1 87 86 22 97; Dr Sylviane Olschwang). The indication for a Latarjet procedure was anterior shoulder instability with a history of at least 1 dislocation, regardless of sports type, activity level, or bone loss. Patients were excluded if they had any concomitant rotator cuff tendon lesions, or previous ipsilateral shoulder surgery. A total of 217 patients (217 shoulders) met the criteria for inclusion.

### Preoperative assessment

The authors retrieved patient demographics, work activity level, and time from injury to surgery, if the accident occurred during sport, type of sport practiced (overhead/non-overhead, contact/non-contact), preoperative dislocation and subluxation episodes, as well as preoperative clinical scores including Western Ontario Shoulder Instability index (WOSI) [24], Shoulder Instability-Return to Sport After Injury index (SIRSI) [16], and Rowe score [36]. Sports such as handball, rugby, basketball, and judo were considered as overhead contact; tennis, ski, badminton, volley as overhead non-contact; football as non-overhead contact; jogging, cycling as non-overhead non-contact.

### Surgical techniques

Depending on surgeon experience, the two techniques were used to perform the Latarjet procedure: a mini-open technique with a commercially available drill guide (Arthrex GmbH, Munich, Germany) and two 4 mm cannulated cancellous screws as based on the modified Latarjet procedure described by Walch and Boileau [38], or an arthroscopic technique with a specific guide (DePuy, Indiana, United States) and two 3.5-mm cannulated cancellous screws as described by Lafosse et al. [26]

### Postoperative rehabilitation

Shoulders were immobilized with a sling for a minimum of 2 weeks to prevent pain, and all patients started self-rehabilitation exercises 1 day after surgery following a standard protocol [34], followed by a rehabilitation program supervised by a physiotherapist. Patients were allowed to return to daily activities after 1 month, but allowed to resume sports after 3 months only if they had recovered mobility. Patients were allowed to RTP if they were pain-free, with full ROM, regardless of time since index surgery.

### Postoperative evaluation

Patients underwent clinical examination at 8 months, during which the authors recorded immobilization time, whether and when a patient returned to their main preoperative sport, as well as postoperative clinical scores including WOSI, SIRSI, and Rowe. The timeframe of 8 months was based on the findings of a systematic review by Hurley et al. [22] who reported that the mean time for RTP following the Latarjet procedure was 5.8 months, ranging from 3.2 to 8 months.

### Statistical analysis

A priori sample size calculation was performed to determine whether the rate of the RTP would be significantly different among overhead athletes vs. non-overhead athletes. From experience with the Latarjet procedure, the proportion of overhead athletes is typically double that of non-overhead athletes. Based on the findings of Baverel et al. [5] and Beranger et al. [6], the expected rates of RTP between two groups of athletes with different characteristics would be 50% in overhead athletes vs. 75% in non-overhead athletes. To determine whether such a difference is statistically significant, with alpha of 0.05 and a power of 0.80, a minimum of 70 overhead athletes and 35 non-overhead athletes is required. The normality of distributions was assessed using Shapiro–Wilk tests. For normally distributed continuous data, differences between groups were evaluated using ANOVA, while for not-normally distributed data, differences were evaluated using Kruskal–Wallis tests. Pre- and postoperative scores were evaluated using paired *t* tests or Wilcoxon signed-rank tests. For categorical data, differences between groups were evaluated using Fisher’s exact tests. Uni- and multivariable regression analyses were performed to determine the influence on rate and time to RTP of 10 independent preoperative variables (age at surgery, BMI, sex, type of sport, dislocation episodes, time from 1st dislocation to surgery, completed rehabilitation program, type of surgery, preoperative scores including WOSI, SIRSI, and Rowe). Statistical analysis was performed using R version 3.4.3 (R Foundation for Statistical Computing, Vienna, Austria). *p* values < 0.05 were considered statistically significant.

## Results

The 217 patients assessed comprised 184 males (85%) and 33 females (15%) aged 26.8 ± 7.3 years (range, 15–62) at index surgery (Table 1). For the majority of patients, the main sport practiced prior to surgery involved overhead (*n* = 173, 80%) and/or contact (*n* = 152, 70%) activities (Table 2). Among 173 overhead athletes, 109 (63%) underwent surgery on their dominant arm. Twenty-seven complications were observed including hematoma (*n* = 23, 11%), graft displacement (*n* = 3, 1.4%), and paresthesia (*n* = 1, 0.5%). Only 140 patients (65%) completed the full rehabilitation program under the supervision of a physiotherapist, while the remaining 77 patients (35%) did not complete the full rehabilitation program.

**Table 1:** Patient demographics

	Cohort *n* = 217 shoulders	
	*n* (%) Mean ± SD	Range
Age at operation (years)	26.8 ± 7.3	(15–62)
BMI (kg/m^2^)	23.8 ± 3.0	(18–39)
Years from first dislocation to surgery	4.4 ± 4.9	(0–26)
Male sex	184 (85%)	
Operation on dominant arm	109 (50%)	
Work activity level		
Active	97 (45%)	
Semi-sedentary	45 (21%)	
Sedentary	75 (35%)	
Type of sport		
Overhead—contact	136 (63%)	
Overhead—non-contact	37 (17%)	
Non overhead—contact	16 (7%)	
Non overhead—non-contact	28 (13%)	
Accident during sport	182 (84%)	
Accident due to		
Fall on shoulder	78 (36%)	
Fall on elbow	20 (9%)	
Movement without fall	77 (35%)	
Contact without fall	39 (18%)	
Unknown	3 (1%)	
Preoperative dislocation episodes		
1	74 (34%)	
2–6	132 (61%)	
7 or more	11 (5%)	
Preoperative subluxation episodes		
1	53 (24%)	
2–6	73 (34%)	
7 or more	91 (42%)	
Hill–Sachs lesion	99 (46%)	
Glenoid anterior defect	103 (47%)

**Table 2:** Postoperative evaluation by preoperative sport type

	Entire cohort		Overhead				Non overhead					*p value*
	*n* = 217		Contact *n*=136		Non-contact *n*=37		Contact *n*=16		Non-contact *n*=28			
	Mean ± SD *n* (%)	Range	Mean ± SD *n* (%)	Range	Mean ± SD *n* (%)	Range	Mean ± SD *n* (%)	Range	Mean ± SD *n* (%)	Range		
Type of Surgery												n.s
Mini-open	196 (90%)		127 (93%)		30 (81%)		14 (88%)		25 (89%)			
Arthroscopic	23 (11%)		9 (7%)		7 (19%)		2 (13%)		3 (11%)			
Immobilization (weeks)	2.4 ± 0.8	(2.0–6.0)	2.4 ± 0.7	(2.0–4.0)	2.6 ± 1.0	(2.0–6.0)	2.3 ± 0.5	(2.0–3.0)	2.3 ± 0.9	(2.0–6.0)		n.s
Completed rehabilitation program	140 (65%)		94 (69%)		21 (57%)		9 (56%)		16 (57%)			n.s
Return to preoperative sport	158 (73%)		99 (73%)	(2.1–8.0)	23 (62%)		14 (88%)		22 (79%)			n.s
Time to return to sport (months)	5.1 ± 1.5	(2.1–8.0)	5.0 ± 1.5		5.3 ± 1.5	(2.6–8.0)	5.2 ± 1.7	(2.9–7.9)	5.2 ± 1.5	(2.6–8.0)		n.s
WOSI score												
Preoperative	55 ± 21	(9–98)	55 ± 21	(9–98)	57 ± 20	(18–95)	53 ± 15	(21–76)	53 ± 22	(14–88)	n.s	
Postoperative	86 ± 13	(31–100)	86 ± 14	(31–100)	88 ± 14	(38–100)	79 ± 17	(51–97)	84 ± 9	(14–88)	n.s	
Net improvement	32 ± 24	(−32–91)	33 ± 25	(−32–91)	31 ± 22	(-30–63)	25 ± 24	(−2–60)	32 ± 20	(14–88)	n.s	
SIRSI score												
Preoperative	51 ± 26	(0–100)	52 ± 27	(0–95)	42 ± 27	(0–100)	51 ± 23	(17–90)	58 ± 23	(20–97)	n.s	
Postoperative	70 ± 23	(8–100)	69 ± 25	(8–100)	78 ± 19	(27–98)	73 ± 17	(42–93)	68 ± 21	(30–100)	n.s	
Net improvement	20 ± 35	(−68–90)	19 ± 36	(-68–90)	32 ± 31	(-15–87)	24 ± 36	(-35–60)	12 ± 33	(-57–57)	n.s	
ROWE score												
Preoperative	52 ± 25	(5–100)	53 ± 26	(5–100)	46 ± 21	(10–100)	51 ± 15	(25–70)	56 ± 26	(15–100)	n.s	
Postoperative	87 ± 16	(15–100)	86 ± 17	(15–100)	89 ± 16	(40–100)	86 ± 14	(60–100)	88 ± 13	(70–100)	n.s	
Net improvement	33 ± 30	(−85–95)	30 ± 33	(−85–95)	41 ± 21	(-5–75)	34 ± 22	(−10–65)	35 ± 25	(0–85)	n.s	

**Fig. 1:**
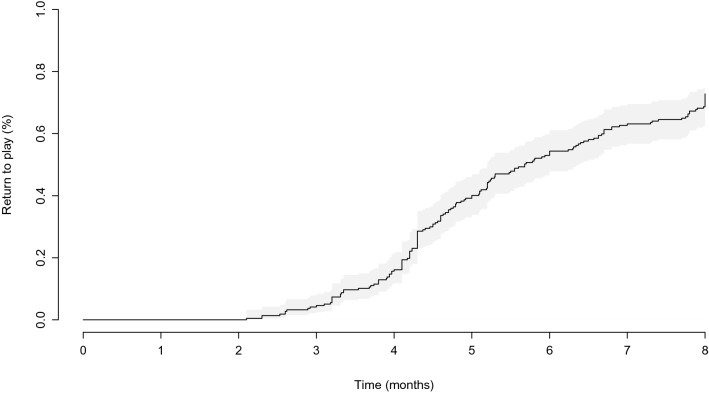
Following the Latarjet procedure, patients started resuming their main sport during the third postoperative month, and by the eight postoperative month, 73% of patients had resumed their main sport

**Fig. 2:**
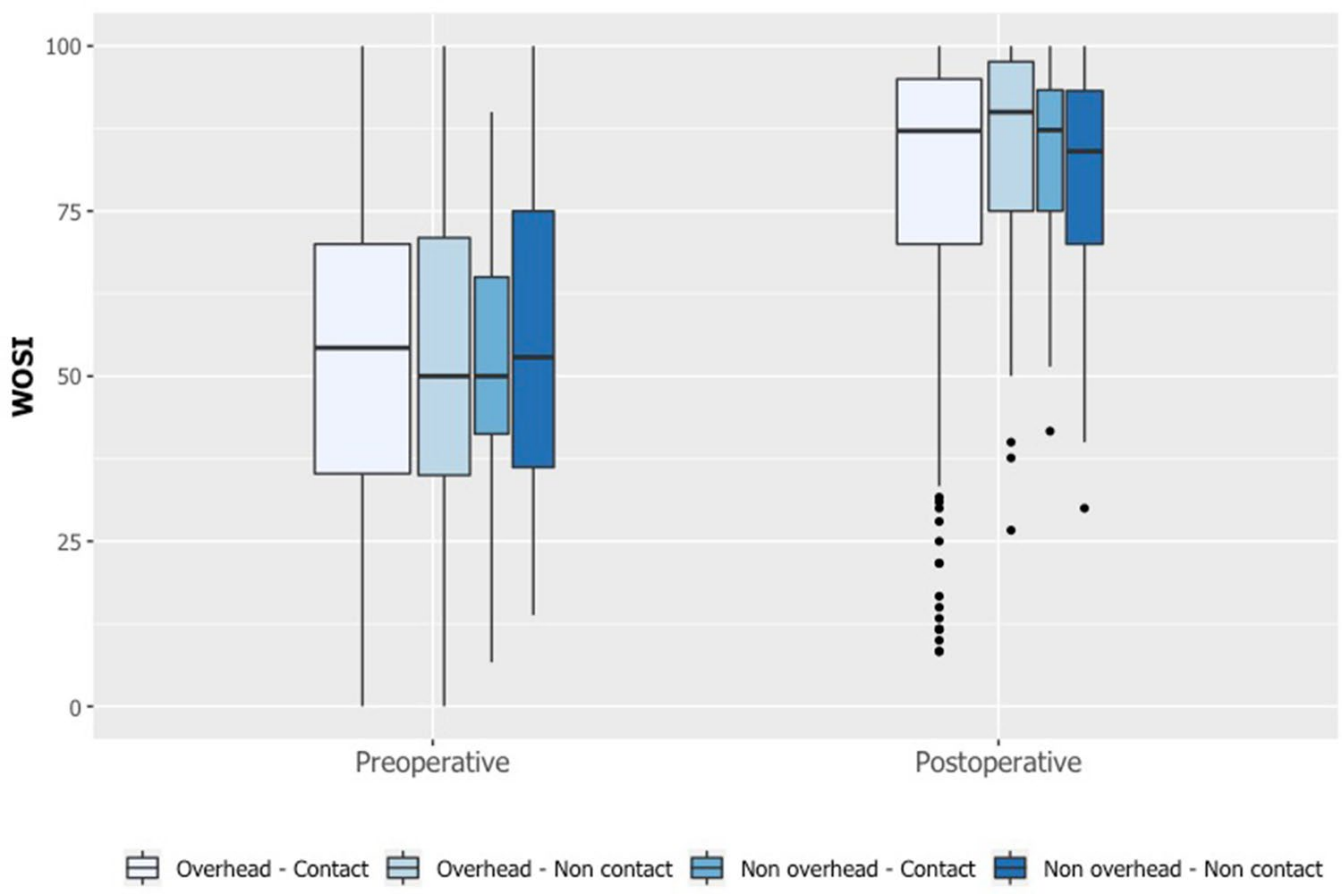
Pre- and postoperative WOSI score by type of sport

By 8 months' follow-up, 158 patients (73%) had resumed their main sport, at a mean of 5.1 ± 1.5 months (Fig. 1). Of the 158 patients that returned to sport, 106 (67%) returned to their preoperative level, while 14 (9%) returned to a higher level, and 38 (24%) returned to a lower level. Patients performing overhead contact had resumed their main sport at a mean of 5.0 ± 1.5 months, overhead non-contact at 5.3 ± 1.5 months, non-overhead contact at 5.2 ± 1.7 months, and non-overhead non-contact at 5.2 ± 1.5 months (n.s.). Comparing pre- and postoperative scores, WOSI improved from 55 ± 21 to 86 ± 13 (*p* < 0.001), SIRSI improved from 51 ± 26 to 70 ± 23 (*p* < 0.001), and Rowe improved from 52 ± 25 to 87 ± 16 (*p* < 0.001). Of the 173 patients that practiced overhead sports, 122 (70%) had resumed their main sport, while of the 44 patients that practiced non-overhead sports, 36 (82%) had resumed their main sport (n.s.). Of the 152 patients that practiced contact sports, 113 (74%) had resumed their main sport, while of the 65 patients that practiced non-contact sports, 45 (69%) had resumed their main sport (n.s.). The type of main sport was not significantly associated with either time for RTP (n.s.), WOSI score (n.s.), SIRSI (n.s.), or Rowe score (n.s.) (Fig. 2).

Univariable analysis revealed that RTP was more likely for patients with higher preoperative Rowe score (OR 1.02, *p* = 0.014), and tended to be more likely for patients with higher preoperative SIRSI score (OR 1.01, n.s.) (Table 3; 4). Multivariable analysis confirmed that RTP was more likely for patients with higher preoperative Rowe score (OR 1.02, *p* = 0.024) and SIRSI score (OR 1.02, *p* = 0.008).

**Table 3:** Uni- and multivariable regression analysis for likelihood of return to sport

	Return to Sport (158 events on 217 followed up shoulders)					
	Univariable			Multivariable (*n* = 209)		
	OR (95% C.I.)		*p* value	OR (95% C.I.)		*p* value
Age	0.97	(0.94–1.01)	*n.s*	0.97	(0.92–1.04)	*n.s*
BMI	1.03	(0.09–16.44)	*n.s*	0.97	(0.86–1.10)	*n.s*
Male sex	1.20	(0.51–2.64)	*n.s*	0.57	(0.18–1.59)	*n.s*
Type of sport						
Overhead—contact	REF					
Overhead—non-contact	0.61	(0.29–1.34)	*n.s*	0.65	(0.23–1.86)	*n.s*
Non overhead—contact	2.62	(0.69–17.17)	*n.s*	2.17	(0.50–15.25)	*n.s*
Non overhead—non-contact	1.37	(0.54–3.96)	*n.s*	0.90	(0.28–3.24)	*n.s*
Dislocation episodes						
1	REF					
2–6	1.10	(0.58–2.06)	*n.s*	1.46	(0.65–3.31)	*n.s*
7 or more	1.06	(0.28–5.17)	*n.s*	2.99	(0.54–25.00)	*n.s*
Time from first dislocation to surgery	1.00	(0.94–1.06)	*n.s*	1.05	(0.96–1.16)	*n.s*
Completed rehabilitation program	− 0.41	(0.34–1.25)	*n.s*	0.68	(0.29–1.54)	*n.s*
Type of surgery						
Latarjet	REF					
Latarjet + anterior capsuloplasty	0.90	(0.50–1.65)	*n.s*	0.73	(0.34–1.56)	*n.s*
Preoperative WOSI	1.01	(0.99–1.02)	*n.s*	0.99	(0.97–1.01)	*n.s*
Preoperative SIRSI	1.01	(1.00–1.02)	*n.s*	1.02	(1.01–1.04)	0.008
Preoperative ROWE	1.02	(1.00–1.03)	0.014	1.02	(1.00–1.04)	0.024

*C.I.* Confidence interval

**Table 4:** Uni- and multivariable regression analysis for time (months) to return to sport

Variables	Return to Sport (158 patients of 217 followed up shoulders)			
	Univariable		Multivariable (*n* = 143)	
	*β* (95% C.I.)	*p* value	*β* (95% C.I.)	*p* value
Age	0.0 (0.7–0.3)	n.s	0.0 (0.0–0.1)	*n.s*
BMI	0.0 (0.0–0.0)	n.s	0.1 (0.0–0.2)	n.s
Male sex	− 0.2 (− 0.9–0.5)	n.s	− 0.5 (− 1.3–0.4)	n.s
Type of sport				
Overhead—contact	REF			
Overhead—non-contact	0.2 (− 0.5–0.9)	n.s	0.4 (− 0.4–1.3)	n.s
Non overhead—contact	0.2 (− 0.7–1.0)	n.s	0.3 (− 0.7–1.3)	n.s
Non overhead—non-contact	0.1 (− 0.6–0.8)	n.s	0.3 (− 0.6–1.1)	n.s
Dislocation episodes				
1	REF			
2–6	0.2 (− 0.3–0.7)	n.s	0.2 (− 0.5–0.8)	n.s
7 or more	0.7 (− 0.4–1.8)	n.s	0.9 (− 0.3–2.1)	n.s
Time from first dislocation to surgery	0.0 (− 0.1–0.0)	n.s	0.0 (− 0.1–0.0)	n.s
Completed rehabilitation program	0.1 (− 0.4–0.6)	n.s	0.0 (− 0.1–0.0)	n.s
Type of surgery				
Latarjet	REF			
Latarjet + anterior capsuloplasty	− 0.2 (0.7–0.3)	n.s	− 0.1 (− 0.7–0.5)	n.s
Preoperative WOSI	0.0 (0.0–0.0)	n.s	0.0 (0.0–0.0)	n.s
Preoperative SIRSI	0.0 (0.0–0.0)	n.s	0.0 (0.0–0.0)	n.s
Preoperative ROWE	0.0 (0.0–0.0)	n.s	0.0 (0.0–0.0)	n.s

*β*, expected difference; *C.I.* confidence interval

## Discussion

**Fig. 3:**
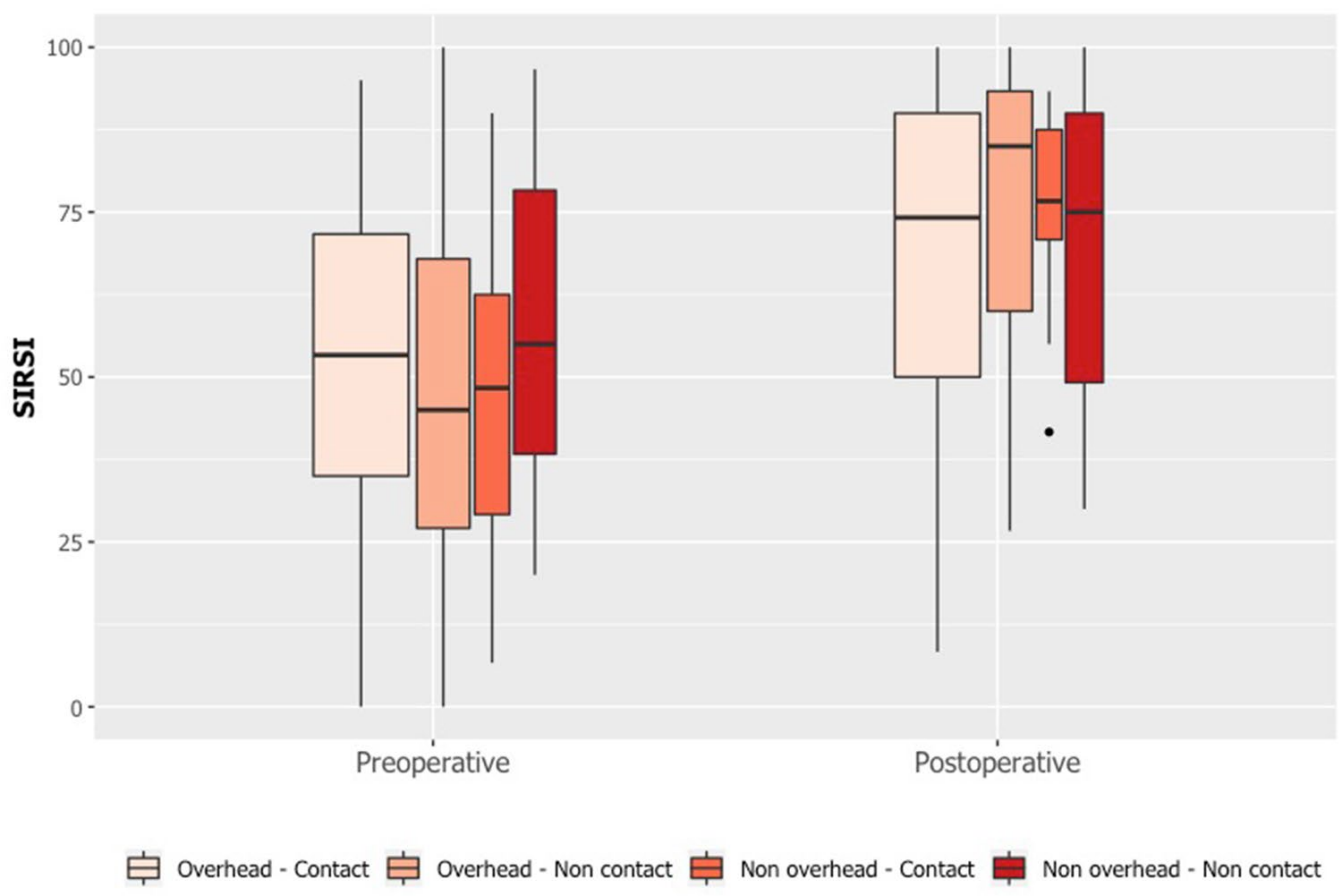
Pre- and postoperative SIRSI score by type of sport

**Fig. 4:**
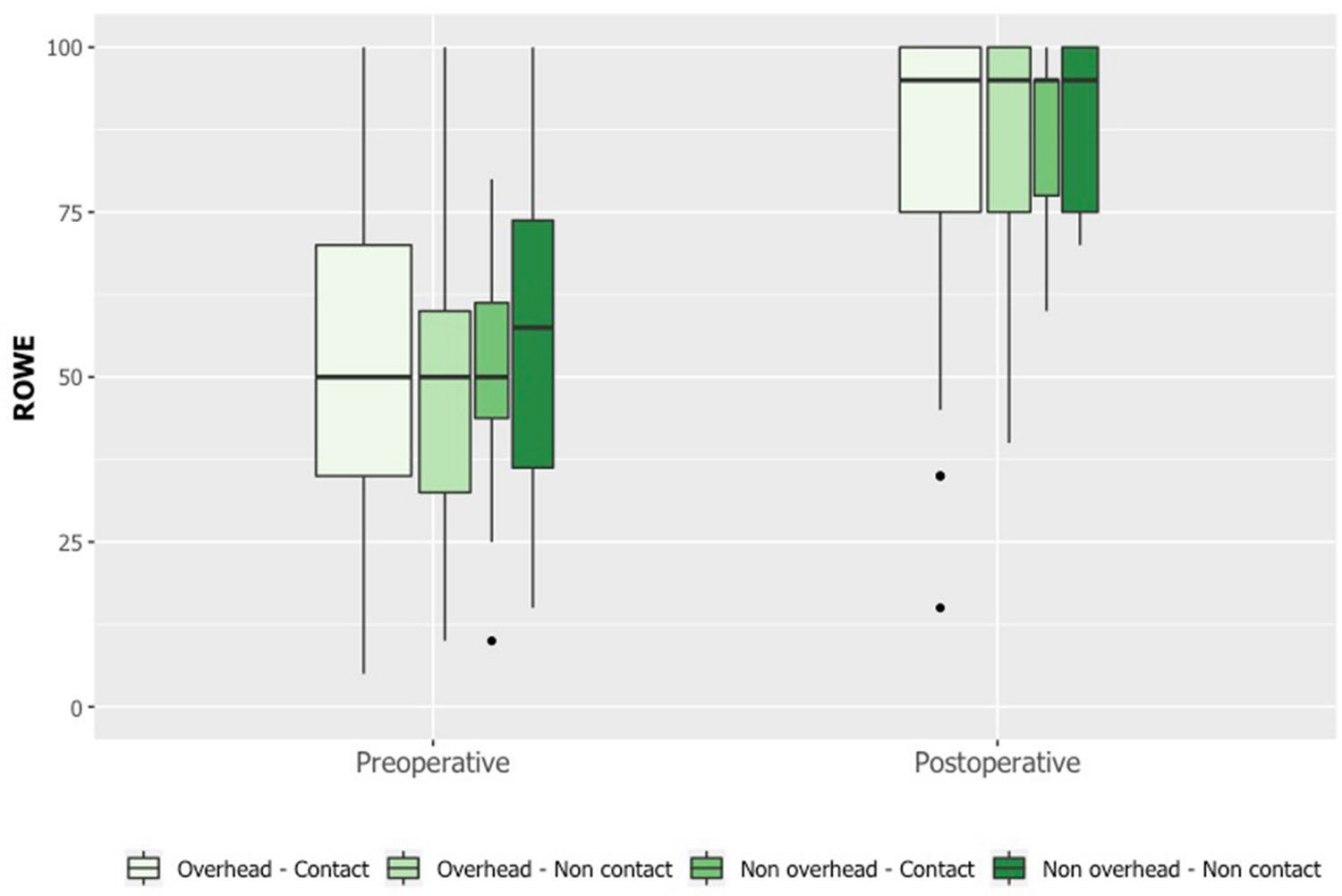
Pre- and postoperative Rowe score by type of sport

The most important findings of this study were that, by 8 months following the Latarjet procedure, 73% of patients resumed their main sport, and that the mean time for RTP was 5.1 ± 1.5 months. The likelihood of RTP was significantly associated with preoperative Rowe and SIRSI scores, but not with the type of sport. The same proportion of patients resumed contact and non-contact sports, while a greater proportion of patients resumed non-overhead sports than overhead sports, although there was no statistically significant difference. Therefore, the present findings do not entirely support the hypothesis that the rate and time of RTP are associated with neither type of sport nor patient characteristics (Fig. 3, 4).

In a systematic review of 31 articles on surgical procedures to treat anterior shoulder instability, Hurley et al. [22] reported that at a mean follow-up of 83.5 months (range, 21–240), 73% of patients resumed their sports at the same or higher level. In another systematic review, Abdul-Rassoul et al. [1] reported that at a mean follow-up of 61 months (range, 12–144), 84% of patients resumed their sports after the Latarjet procedure, the majority of which returned to their preinjury level. Privitera et al. [30] reported that at a follow-up of 52 months (range, 24–120), 49% of patients returned to their competitive sport at their preinjury level, and 14% of patients returned to their competitive sport at a decreased level. Rossi et al. [33] revealed that at a follow-up of 58 months (range, 24–108 months), 96% of patients were able to return to sports, of which 91% returned to their preinjury level. Baverel et al. [5] found that at a mean follow-up of 46 months, almost twice as many competitive athletes (79%) returned to their preinjury level or higher, compared to recreational athletes (43%). Even though the Latarjet procedure does decrease shoulder mobility, it has minimal impact on RTP [35], and may decrease progression of osteoarthritis [37].

In the present series, the mean time for RTP was 5.1 ± 1.5 months, with no significant differences among the 4 types of sports. Rossi et al. [33] reported that time for RTP after the Latarjet procedure was 3.7 months for patients who performed non-contact/non-overhead sports, compared to 5.1 months for those that performed high-impact/contact sports, 5.4 months for overhead sports, and 5.8 months for martial arts. Frantz et al. [14] found that at 6 months follow-up, 55% of patients failed to meet criteria for RTP, defined as < 20° loss of ROM as compared with baseline values in any plane, and/or lower strength grade than the baseline value. Several systematic reviews report return to play (RTP) within 5.8 months following the Latarjet procedure [21, 28], but the type of sport and preoperative patient characteristics that influence RTP remain unknown.

In the present study, uni- and multivariable analyses revealed that patients with better preoperative Rowe and SIRSI scores were more likely to resume their main sport. Gerometta et al. [16] reported that the patient’s psychological readiness should be taken into account when deciding on RTP [2]. The SIRSI score, like the Anterior Cruciate Ligament–Return to Sport after Injury (ACL-RSI) score [7], enables identification of patients who might have psychological difficulties returning to play. A recent systematic review by Ardern et al. [4] identified studies evaluating the psychological factors associated with RTP, which include motivation, self-confidence, and fear. Fear is the main negative emotion influencing RTP, and a common reason for patients not to resume sports. Self-determination theory ensures that the patients perceive that they are in control of resuming sports, by reducing external pressure and overcoming fear by involving the patients in setting rehabilitation as well as RTP goals, and therefore promoting their sense of confidence and autonomy.

The results of the present study must be interpreted with the following limitations in mind. This was a retrospective multicentric study that included 9 centers, which may have introduced multiple confounding factors, such as different surgical techniques, including a majority of mini-open procedures (*n* = 196), and a minority of arthroscopic procedures (*n* = 21). Furthermore, the numbers of patients excluded because of concomitant rotator cuff tears or surgical antecedents on the ipsilateral shoulder was not documented. This study, with its short follow-up, was not designed to determine the recurrence of dislocations after the Latarjet procedure or to report radiological outcomes, but rather to quantify the rate and time for RTP within 8 months regarding different types of sport. It is worth noting that the rate and mean time for RTP could both be greater if the study was performed at longer follow-up. Nevertheless, the present series is one of the largest cohorts on outcomes following the Latarjet procedure, which enabled detailed analysis of independent variables that could improve or compromise rate of RTP. The present findings could help surgeons provide objective advice to patients regarding resuming sports after the Latarjet procedure, depending on sport type and preoperative scores.

## Conclusion

The most important findings of this study were that, by 8 months following the Latarjet procedure, 73% of patients resumed their main sport, and that the mean time for RTP was 5.1 ± 1.5 months. The likelihood of RTP was significantly associated with preoperative Rowe and SIRSI scores, but not with the type of sport. The same proportion of patients resumed contact and non-contact sports, while a greater proportion of patients resumed non-overhead sports than overhead sports, although there was no statistically significant difference.
